# Intramolecular vibrations enhance the quantum efficiency of excitonic energy transfer

**DOI:** 10.1007/s11120-020-00742-x

**Published:** 2020-04-18

**Authors:** Hong-Guang Duan, Peter Nalbach, R. J. Dwayne Miller, Michael Thorwart

**Affiliations:** 1 I. Institut für Theoretische Physik, Universität Hamburg, Jungiusstraße 9, 20355 Hamburg, Germany; 2 Max Planck Institute for the Structure and Dynamics of Matter, Luruper Chaussee 149, 22761 Hamburg, Germany; 3 The Hamburg Center for Ultrafast Imaging, Luruper Chaussee 149, 22761 Hamburg, Germany; 4 Westfälische Hochschule, Münsterstr. 265, 46397 Bocholt, Germany; 5 The Departments of Chemistry and Physics, University of Toronto, 80 St. George Street, Toronto, M5S 3H6 Canada

**Keywords:** Excitation energy transfer, Vibronic coupling, Efficiency of exciton transfer

## Abstract

We study the impact of underdamped intramolecular vibrational modes on the efficiency of the excitation energy transfer in a dimer in which each state is coupled to its own underdamped vibrational mode and, in addition, to a continuous background of environmental modes. For this, we use the numerically exact hierarchy equation of motion approach. We determine the quantum yield and the transfer time in dependence of the vibronic coupling strength, and in dependence of the damping of the incoherent background. Moreover, we tune the vibrational frequencies out of resonance with the excitonic energy gap. We show that the quantum yield is enhanced by up to 10% when the vibrational frequency of the donor is larger than at the acceptor. The vibronic energy eigenstates of the acceptor acquire then an increased density of states, which leads to a higher occupation probability of the acceptor in thermal equilibrium. We can conclude that an underdamped vibrational mode which is weakly coupled to the dimer fuels a faster transfer of excitation energy, illustrating that long-lived vibrations can, in principle, enhance energy transfer, without involving long-lived electronic coherence.

The first steps of photosynthesis involve a quantum mechanical transfer of excitation energy between different spatial regions of a molecular complex. The understanding of the fundamental mechanisms of the transfer process is still an important and ongoing challenge to fundamental science, especially in view of the role of quantum coherence for the transfer efficiency (Scholes et al. 2011; Marais et al. 2018). The latter involves the quantum dynamics of excited electronic states which strongly interact with vibrational degrees of freedom of the molecular host (Hestand and Spano 2018) and the dielectric environment of the molecule. Since modern ultrafast non-linear optical spectroscopy opens a window to observe time scales of dynamic processes from the femtosecond to the picosecond regime, a growing number of works has considered the non-equilibrium quantum dynamics of the excitons and a clear picture has emerged in recent years.

Experimentally observed coherent beating signals in optical 2D spectra are only attributed to electronic coherences on very short times scales (~ 50 fs). Beatings on longer times scales (ps) are then attributed to vibrational coherence (Halpin et al. 2014; Chin et al. 2013; Nalbach and Thorwart 2012; Duan et al. 2015a) which shows up in optical spectra due to a typically weak vibronic coupling. The main obstacle to long-lived electronic quantum coherence is certainly the dissipative environment which is formed by the vibrational motion of the many nuclei constituting the molecular backbone and by the strong dielectric response of the aqueous solvent in which the molecules are embedded. This commonly leads to significant fast quantum mechanical dephasing and relaxation of the electronic degrees of freedom (Duan et al. 2017; Thyrhaug et al. 2018). Yet discrete molecular vibrational modes may exist which interact with the electronic sector and can, in principle, influence the efficiency of transfer of electronic excitation energy.

Vibrational coherences are mostly influenced by the intramolecular potential with only weak couplings to the bath, which manifests itself in vibrational coherence times being much longer than electronic coherence times and commonly exist for several picoseconds (Duan et al. 2015a). Yet the vibrational amplitudes are usually small, do not rely on quantum superposition states, and are thus a standard phenomenon of classical mechanics. It is, however, an interesting question how electronic and vibrational coherence collude and whether vibrational coherence is in principle able to influence the efficiency of the transfer of electronic excitation energy, i.e., the quantum yield or the transfer times, and whether the interplay is significant for biological functionality.

Since electronic dephasing is much faster than the exciton transfer time, dynamic electronic coherence (as opposed to static coherence involving the overlap of electronic wave functions) is considered irrelevant for the transfer and only vibrational coherence is a candidate for influencing the transfer efficiency which occurs on time scales similar to the life time of vibrational coherence of a few picoseconds. At time scales beyond the electronic dephasing time, the occupation probability of the pigment site within a light-harvesting complex will essentially be thermal. Then, inter-complex energy transfer, i.e., from a light-harvesting antenna complex to a reaction center, is determined by the coupling to the reaction center (typically much smaller than the intra-complex coupling) and the thermal occupation of those pigments which are most strongly coupled to the reaction center. Thus, clearly, the time dependence of the occupation of the exit site of the network (or the acceptor) is the relevant quantity to determine the overall quantum yield of the transfer. It has also been argued that vibrational coherence acts also as a resource to promote energy transfer to the exit site, especially, when the vibrational frequency is in resonance with the excitonic energy gap of the involved exciton states (Novoderezhkin et al. 2015; Maly et al. 2016; Novoderezhkin et al. 2017; Renaud and Grozema 2015; Bakulin et al. 2015; Falke et al. 2014; Narzi et al. 2016).

The role of intramolecular vibrational modes in the exciton transfer has been studied a lot in the context of the Fenna Matthews Olson (FMO) protein (Nalbach and Thorwart 2012; Nalbach et al. 2015; Mujica-Martinez and Nalbach 2015; Schulze and Kühn 2015; Schulze et al. 2016; Butkus et al. 2014), which is a rather high-level molecular complex, the monomer of which consists of eight excitonic sites. The coupling of the exciton states to an underdamped vibrational mode in the FMO complex has been shown to play a more subtle role. A model study has revealed (Nalbach et al. 2015) that only when all excitonic sites considered are coupled to a vibrational mode, or the exit site 3 only is coupled to its own oscillator mode, the transfer time is reduced. When intermediate electronic sites occupied along the transfer pathway from entrance to exit are coupled to a single underdamped vibrational mode, the energy typically gets trapped in those vibrational modes during its way through the network of sites. This reduces the transfer efficiency considerably. These model calculations apply to the seven-site FMO complex, a study of the role of the vibrational coupling on the basis of the most simple model of a dimer has been lacking up to present. It is the major goal of the present work to close this gap.

In the context of the interplay with electronic coherence, it was argued that nature exploits long-lived coherent vibrations to induce long-lived electronic coherence in excitons (Chin et al. 2013) when the vibrational and electronic degrees of freedom are resonantly coupled. The vibrational mode thereby has been suggested to act as a “phonon laser” on the excitons and to induce ultralong electronic coherence exisiting on a time scale of the vibrational coherence (Plenio et al. 2013). Yet a close inspection of the full FMO model with realistic bath spectral densities reveals (Nalbach and Thorwart 2012) that this mechanism is not realistic due to the presence of strong dissipation in all channels.

Moreover, an interesting mechanism has been suggested by Tiwari et al. (2013, 2017) in which non-adiabatic electronic-vibrational coherent mixing at short times resonantly enhances the amplitude of the particular delocalized anticorrelated vibrational mode on the ground electronic state. This concept does not involve long-lived electronic coherence and is in general in agreement with the observation that a strong vibronic coupling produces large-amplitude coherent oscillations of the electronic component with a usual short lifetime and a long-lived vibrational coherence albeit with a rather small amplitude (Duan et al. 2015a; Halpin et al. 2014). Yet while the mechanism has been proposed for an isolated quantum system, the role of fluctuations needs to be addressed as well, with an explicit coupling to an electronic and a vibrational bath (Yeh et al. 2018). The concept that an increased vibronic coupling survives weak electronic dephasing at short times and induces a resonantly enhanced long-lived vibrational coherence of the anticorrelated mode was shown to apply, however, only when the electronic coherence is weakly dephased. For more realistic stronger electronic dephasing [as measured in the experiment by Duan et al. (2017)], the required long electronic coherence times are absent and the mechanism is not realistic (Duan et al. 2019). However, the impact on the transfer efficiency has not been addressed in these works.

A number of papers have addressed the interplay of electronic and vibrational dynamics in different contexts. A reduced 3-site model has been used by Irish et al. (2014) to show that excited vibrational states on the excited electronic manifold can create resonant pathways through the system, supporting fast and efficient transfer. The resonance condition was identified as crucial for the significant enhancement of the efficiency and directionality of energy transport. Moreover, a dimer in the presence of classical colored noise has been considered by Dijkstra et al. (2015). This model corresponds to the coupling of a dimer to a classical vibrational mode in the presence of non-Markovian vibrational damping. An optimal transfer rate has been found for an intermediate damping regime and when the electron-vibrational coupling is resonant.

The enhancing impact of vibrational modes on quantum transport is not limited to molecules in solution, but can also occur in the solid-state phase. Semião et al. (2010) have analyzed the exciton transfer along a chain of sites in a conjugated polymer when all sites couple to a common nanomechanical resonator mode which is also externally driven. The transfer efficiency can be tuned by tuning the driving frequency of the nanoresonator.

In the present work, we analyze the functionality and the impact of underdamped vibrations on the transfer efficiency in a minial model and without the inclusion of many other molecular details which might obscure the picture. For this, we study an excitonically coupled donor-acceptor pair, in which each partner is coupled to its own underdamped vibrational mode and, in addition, to a continuous background of environmental solvent modes. A key aspect of our work is the use of experimentally measured and, thus, realistic parameters in the model. To describe the exciton dynamics in the dimer numerically exactly, we use the hierarchy equation of motion (HEOM) approach (Ishizaki and Tanimura 2005). We study the quantum yield in the form of the asymptotic occupation probability of the acceptor site depending on the Huang-Rhys factor, describing the coupling strength between the electronic and vibrational states, and its dependence on the damping strength due to the incoherent background. In addition, we determine the transfer time of the population from donor to acceptor. We find that the transfer is in general faster for weaker vibronic coupling. The quantum yield is, however, almost independent of the damping due to the continuous bath as well as from the Huang-Rhys factor of the vibrational modes. In contrast, we observe that the quantum yield is larger by up to 10% when the frequency of the vibrational mode coupled to the acceptor is smaller than the frequency of the vibrational mode at the donor.

Vibrational modes at an excitonic site create a ladder of available energy levels. Our findings show that energy levels closer in energy at the acceptor as compared to the donor resulting in an increased quantum yield. This effect can be understood in that the coupling to the vibrations yields a shift in the energy levels and, by this, provides a condition closer to resonance between the donor and acceptor given the site energy difference. Thus, we can conclude that the efficiency of the energy transfer can be tuned by coupling the dimer, in particular the acceptor, to underdamped vibrational modes. Moreover, this also shows clearly that long-lived electronic coherence is of no relevance for the vibrational promotion of the transfer.

Recently, a related model has been studied by Wang and Zhao (2019) who have also investigated the impact of a vibrational mode on energy transfer. Apart from general similarities in the methodology, fundamental differences between the work of Wang and Zhao and this work have to be pointed out. A crucial difference is that we consider the configuration that the donor and the acceptor states couple to their own vibrational mode with different vibrational frequencies. In contrast, Wang and Zhao have started with a dimer whose individual electronic states are coupled to the same single vibrational mode. This is crucial since a coupling to the same mode can induce artificial quantum entanglement or vibronic coupling between donor and acceptor which is absent in the case of two independent modes. A further difference is that we consider the usual Holstein-type coupling of the electronic states to the vibrational mode, while Wang and Zhao introduce also an off-diagonal Peierls-type vibrational coupling characterized by another free model parameter. From our analysis, in terms of the polaron transformation (see below), a Holstein-type coupling to two independent modes also introduces a Peierls-type coupling of the dimer, i.e., a modulation of the electronic donor-acceptor coupling by the vibrational mode. This effect would mix with the Peierls coupling and can no longer be singled out in the dynamics. A third difference is that we have included two separate channels of dissipation for the electronic states. On one hand, in our model, the donor and acceptor states are coupled to their own direct bath, where the coupling strength is given by *η*. On the other hand, the vibrational modes are separately damped by a “vibrational” bath with a coupling strength *η*_0_. In contrast, Wang and Zhao only consider an electronic bath while the vibrational modes are undamped. A more realistic description, however, requires a vibrational bath to be included. Finally, it is important to realize that we examine a very different mechanism as compared to that studied by Wang and Zhao. We study the energy transfer in the donor-acceptor system by monitoring the population dynamics with a varying vibronic coupling strength and/or the vibrational frequencies of the two coupled modes. Only in a model with two independent vibrational modes such a scenario can be analyzed. This also includes the possibility to vary the number of vibronic transfer channels, as illustrated in Fig. 1. Subsequently, this allows us to study the impact of vibrational coherence on the excitonic energy transfer in the resonant and non-resonant cases. In contrast, the work of Wang and Zhao exclusively focuses on the resonant energy transfer. While the results of both works cannot be compared on the quantitative level due to the mentioned differences, both works use (different) numerically exact approaches to show under different circumstances that the promotion effect of an underdamped vibrational mode is significant only when the excitonic coupling is smaller than the site energy difference.

## Theoretical model

In order to show the principle at work, we consider a dimer model of a coupled donor-acceptor system to investigate the influence of underdamped vibrational modes on the exciton transfer dynamics. Typically, the difference of site energies between two pigments is of the order of 200 cm^−1^ and the electronic coupling $\lesssim$ 100 cm^−1^. To be specific, we choose the donor (D) and acceptor (A) site energies as $\epsilon _{D}=100$ cm^−1^ and $\epsilon _{A}=0$ cm^−1^, and the electronic coupling *J* for a dimer with the molecular Hamiltonian

1 $$H_{{{\text{mol}}}} = |D\rangle \epsilon_D \langle D| + |A\rangle \epsilon_A \langle A| + |A\rangle J\langle D| + H.c.$$

In natural light-harvesting systems, the pigments are usually embedded in a protein skeleton and the whole complex is immersed in water as a solvent. The vibrational modes of the protein and the dielectric modes of the polar solvent act as sources of electrostatic fluctuations on the exciton degrees of freedom (Nitzan 2006). We model them as a bath of harmonic oscillators with the Hamiltonian

2 $$H_{B}=\sum _{m=D,A}\sum _{j}\left( \frac{p^{2}_{m,j}}{2}+\frac{\omega _{m,j}}{2}x^{2}_{m,j} \right) \, .$$

Therein, the $p_{m,j}$ are the momenta and $x_{m,j}$ are the displacement operators of the *j*th bath mode at the pigment *m*. We assume a linear coupling between the exciton states of the pigment and the bath resulting in

3 $$H_{{{\text{int}}}} = \sum\limits_{{m = D,A}} {\sum\limits_{j} {c_{{m,j}} } } x_{{m,j}} |m\rangle \langle m| .$$

The total Hamiltonian is given by

4 $$H_{tot}=H_{mol}+H_{B}+H_{int} .$$

We neglect possible small spatial correlations between the two baths (Nalbach et al. 2010; Olbrich et al. 2011; Aghtar et al. 2013; Rivera et al. 2013). We assume that the bath spectral density consists of two parts, a broad background continuum $J_{\text{back}}(\omega)$ describing solvent modes and a part with pronounced peak at a frequency $\omega _{0,A/B}$ which is rather narrow and which describes an underdamped vibrational mode. For simplicity, we assume a Debye spectral density for the background, i.e., $J_{\text{back}}(\omega )=\frac{\eta \omega \gamma ^{2}}{\omega ^{2}+\gamma ^{2}}$ with the damping constant $\eta$ and the Debye cutoff frequency $\gamma$. In the following, we employ typical parameters, i.e., $0.5\le \eta \le 2$ and $\gamma =200$ cm^−1^, as extracted from experiments with the light-harvesting complex LHCII (Duan et al. 2015b). For the vibrational mode, we include one additional underdamped mode, i.e., a narrow peak, with the width $\gamma _0$ assumed equal for donor and acceptor. The latter describes the weak damping of the vibrational mode itself. Thus, the total spectral density has the form

5 $$J_{{D/A}} (\omega ) = \frac{{\eta \omega \gamma ^{2} }}{{\omega ^{2} + \gamma ^{2} }} + \frac{{\eta _{0} 4\gamma _{0}^{2} \omega _{{D/A}}^{2} \omega }}{{(\omega ^{2} - \omega _{{D/A}}^{2} )^{2} + 4\gamma _{0}^{2} \omega ^{2} }}$$

and the total reorganization energy is

6 $$\lambda =\frac{\eta }{2}\gamma +\eta _{0}\gamma _{0}\, .$$

The Huang–Rhys factor is then given by

7 $$S=\frac{\eta _{0}\gamma _{0}}{\omega _{D/A}}\, .$$

We use the same form of the bath spectra for both pigments and vary only the peak frequencies $\omega _{D/A}$.

In detail, we study a dimer with intermediate electronic coupling $J=50$ cm^−1^. We vary the coupling strength between the continuum background and the system between $0.5\le \eta \le 2$. The cutoff frequency of the background Debye bath spectrum is fixed at $\gamma =200$ cm^−1^. The Huang–Rhys factor of the underdamped mode is varied between $0\le S \le 1$. In addition, we vary the frequencies of the two underdamped intramolecular modes $\omega _{D/A}$. Throughout the paper, we work at room temperature (300 K).

### Mapping to a dissipative dimer-plus-oscillator system

**Fig. 1:**
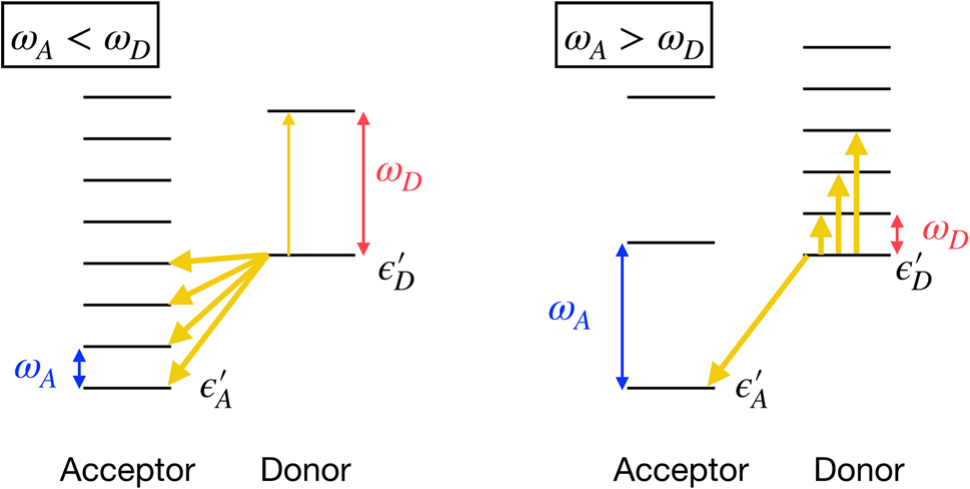
Sketch of the energy diagram of the system Hamiltonian $H^\prime$ after a polaron transformation for the case $\omega _A<\omega _D$ (left) and $\omega _A>\omega _D$ (right). The orange arrows indicate schematically the transitions out of the lowest vibrational level of the donor

Although we are using the above model of a dimer coupled to a bath with the structured spectral density Eq. (5) for all numerical calculations below, it is instructive for the interpretation of the numerical results to express the total Hamiltonian in a different form. For this, we take out the two vibrational modes coupled to the donor and the acceptor of the vibrational bath and include them into the system part (Thorwart et al. 2004). With the bosonic operators *d* and *a* for the donor and acceptor vibrational mode and the coupling constants $g_D$ and $g_A$, we obtain

8 $$\begin{aligned} H_{tot}= & \, H_{mol}+ \omega _D d^\dagger d + \omega _A a^\dagger a+ g_D(d^\dagger +d)|D\rangle \langle D| \nonumber \\&+ g_A(a^\dagger +a)|A\rangle \langle A| \nonumber \\&+ H_{B,el}+H_{B,vib}+ H_{int,el}+H_{int,vib} \, , \end{aligned}$$

where the Hamiltonians of the electronic and the vibrational baths are given by

9 $$H_{B,el}=\sum _k \nu _k b_k^\dagger b_k \, , H_{B,vib}\sum _j \omega _j u_j^\dagger u_j \, ,$$

and the system-bath Hamiltonians are

10 $$\begin{aligned} H_{int,el}= & {} \sum _k (b_k^\dagger +b_k)g_k(|D\rangle \langle D|+|A\rangle \langle A|) \, , \nonumber \\ H_{int,vib}= & {} \sum _j (u_j^\dagger +u_j)[h_{Dj}(d^\dagger + d)+h_{Aj}(a^\dagger + a)] \, . \end{aligned}$$

We have defined the bosonic operators $b_k$ and $u_j$ for the electronic and the vibrational bath, respectively, together with the respective coupling constants $g_{D/A k}$and $h_{D/Aj}$. The spectral densities follow as (Thorwart et al. 2004)

11 $$\begin{aligned} J_{el}(\omega )= & {} \sum _k g_{k}^2 \delta (\omega -\nu _k) = J_{\text{back}}(\omega )\, , \nonumber \\ J_{vib,D/A}(\omega )= & {} \sum _j h_{D/Aj}^2 \delta (\omega -\omega _j) = \kappa _{D/A} \omega e^{-\omega /\omega _c}\, . \end{aligned}$$

We assume the same cutoff frequency $\omega _c$ for both vibrational baths. By comparing the prefactors (Thorwart et al. 2004), we find that

12 $$\begin{aligned} \kappa _{D/A}= & {} \frac{\gamma _0}{\pi \omega _{D/A}} \, , \nonumber \\ g_{D/A}= & {} \frac{1}{2}\sqrt{\pi \eta _0\gamma _0 \omega _{D/A}} \nonumber \\= & {} \frac{ \omega _{D/A}}{2}\sqrt{\pi S}\, . \end{aligned}$$

Next, we use a polaron transformation (Xu and Cao 2016)

13 $$U=e^{\frac{g_D}{\omega _D}(d^\dagger -d)|D\rangle \langle D|+\frac{g_A}{\omega _A}(a^\dagger -a)|A\rangle \langle A|} \, .$$

The transformed Hamiltonian follows as

14 $$\begin{aligned} H^\prime _{tot}= & {} UH_{tot}U^\dagger \nonumber \\= & {} \left[ \epsilon ^\prime _{D} - 2\frac{g_D}{\omega _D}\sum _j h_{Dj}(u_j^\dagger + u_j) \right] |D\rangle \langle D| + \omega _D d^\dagger d \nonumber \\&+\left[ \epsilon ^\prime _{A} - 2\frac{g_A}{\omega _A}\sum _j h_{Aj}(u_j^\dagger + u_j) \right] |A\rangle \langle A| + \omega _A a^\dagger a \nonumber \\&+ J\left[ |A\rangle \langle D| e^{-\frac{g_D}{\omega _D}(d^\dagger -d)+\frac{g_A}{\omega _A}(a^\dagger -a) }+h.c.\right] \nonumber \\&+ H_{B,el}+H_{B,vib}+ H_{int,el}+H_{int,vib} \, , \end{aligned}$$

with the shifted electronic energies

15 $$\epsilon ^\prime _{D/A}= \epsilon _{D/A} - \frac{g_{D/A}^2}{\omega _{D/A}} = \epsilon _{D/A} - \frac{\pi }{4} \eta _0\gamma _0\, .$$

Hence, the vibrational shift of the electronic energies is the same for the donor and the acceptor site when we assume that both vibrational modes couple to their respective site with a strength $\eta _0$. The resulting energy level diagram of the transformed system Hamiltonian is shown in Fig. 1. The coupling to a vibrational mode induces a vibronic coupling described by the term $J\left[ |A\rangle \langle D| e^{-\frac{g_D}{\omega _D}(d^\dagger -d)+\frac{g_A}{\omega _A}(a^\dagger -a) }+h.c.\right]$. For the case $\omega _A<\omega _D$, several vibrational acceptor states lie below the vibrational ground state of the donor and are, thus, energetically favored in a transfer starting from the donor. They offer many transfer channels. In the same way, the vibrational relaxation in the donor, which is described by $\kappa _{D} = \frac{\gamma _0}{\pi \omega _{D}}$, is slower than that in the acceptor, which is determined by $\kappa _{A} = \frac{\gamma _0}{\pi \omega _{A}}>\kappa _{D}$. For the case $\omega _A>\omega _D$, much fewer vibrational acceptor states lie below the vibrational ground state of the donor, hence offering much fewer energetically favored acceptor states for a transfer starting from the donor. In turn, vibrational relaxation in the donor is much faster because $\kappa _{D} > \kappa _{A}$. The consequences for the quantum yield and the transfer times are discussed further below in the section “Dependence on the vibrational frequency”.

Moreover, a stronger coupling of the donor to its vibrational mode increases $g_D / \omega _D$ and thus decreases the electronic coupling *J*, thus decelerating the energy transfer. The energy is used to excite vibrational levels of the donor. Likewise, a stronger coupling of the acceptor to its vibrational mode increases $g_A / \omega _A$ and thus decreases the relative electronic coupling *J* to the acceptor ground state. Thus, during the energy transfer from the donor to the acceptor, vibrational acceptor states are excited and it takes longer for the acceptor to equilibrate, thus slowing down the overall energy transfer. This will be observed below.

**Fig. 2:**
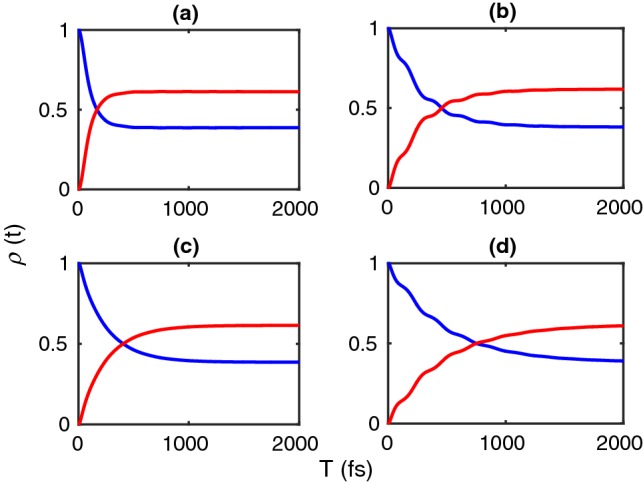
The population dynamics of the donor and the acceptor in the site basis under the influence of a bath with the spectral density given in Eq. (5). The parameters are in all panels $\gamma =200$ cm^−1^, the vibrational frequency $\omega _{0}=142$ cm^−1^, and the linewidth $\gamma _{0}=10$ cm^−1^ of the underdamped mode coupled to the dimer. Moreover, we set in **a**$\eta =0.5$ and the Huang–Rhys factor $S=0.5$, in **b**$\eta = 0.5, S = 1.0$, in **c**$\eta = 2.0, S = 0.02$, and in **d**$\eta = 2.0, S= 1.0$

## Method

We calculate the population dynamics of the dimer model by using the hierarchy equation of motion (HEOM) for the Hamiltonian of Eq. (4). The details of the derivation are described in greater details by Ishizaki and Tanimura (2005) and by Tanaka and Tanimura (2009) and are not reproduced here. In general, the reduced density matrix of the dimer system is obtained by integrating out the bath, starting from a factorized initial state regarding system and bath. The bath initial state is given by a canonical thermal distribution which also includes a thermal initial state of the vibrational modes. By defining auxiliary density matrices, the main equation of motion can be obtained by solving a hierarchy of coupled differential equations. By increasing the depth of the hierarchy until numerical convergence is found, this method provides numerically exact results. The global fitting approach is then applied for the analysis of the energy transfer dynamics from the donor to the acceptor (Prokhorenko 2012). It is important to realize that the presence of the underdamped modes in the bath spectrum induces significant correlations in the time domain and the time correlation function decays in a damped oscillatory way with two frequencies present. The frequencies of these oscillations are determined by the vibrational frequencies $\omega _{D/A}$ and the decay time constants by the width $\gamma _{0}$ of the underdamped modes. Thus, in general, the resulting exciton dynamics is highly non-Markovian and calls for using a numerically exact method such as HEOM. In particular, a Markovian type of quantum master equation is not applicable in this context.

## Time-dependent populations of the donor and the acceptor

**Fig. 3:**
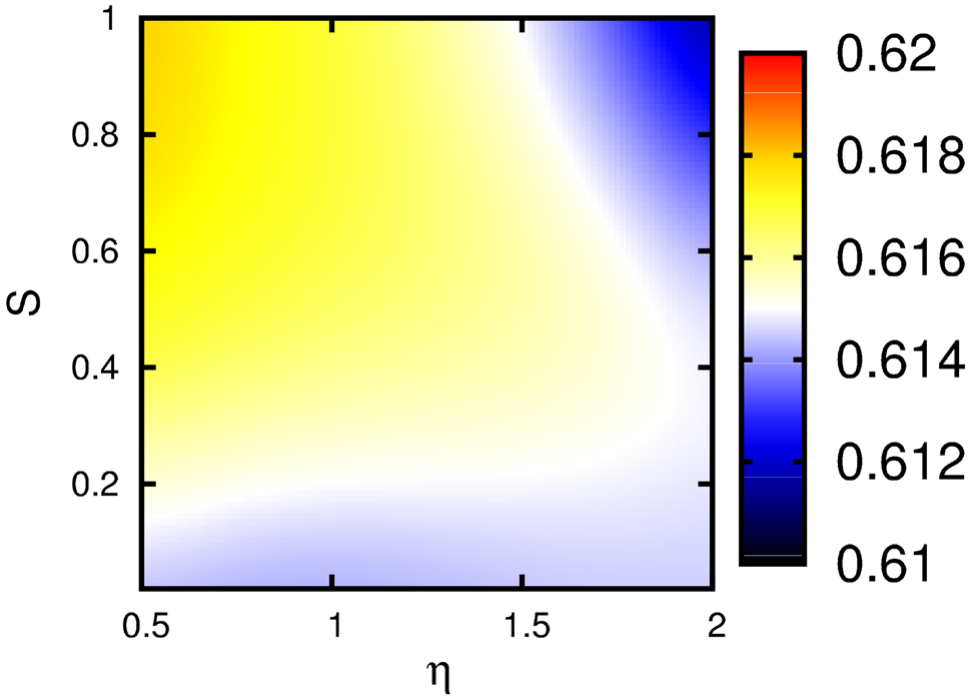
Quantum yield, or, equivalently, the asymptotic population of the acceptor after 2 ps, versus system-background bath coupling strength $\eta$ and the Huang–Rhys factor *S* at temperature 300 K with $\gamma _{0}=10$ cm^−1^

First, we focus on a dimer with underdamped vibrational modes with equal frequencies $\omega _{0}\equiv \omega _{D}=\omega _{A}=142$ cm^−1^. The vibrational frequencies are close to being in resonance with the excitonic gap (neglecting renormalization effects due to the environmental fluctuations). We calculate the time-dependent populations of the dimer states for various Huang–Rhys factors $0\le S\le 1$ and system-bath interaction strengths $0.5\le \eta \le 2$. We assume a small linewidth $\gamma _{0}=10$ cm^−1^ of the underdamped vibrational modes, which ensures long-lived vibrational coherence. The character of the electronic damping is, in general, of overdamped nature and the electronic coherence is very short lived.

Figure 2 shows the population dynamics of the donor and the acceptor in the site basis for different choices of parameters as indicated. For these realistic parameters, no electronic coherent oscillations are observable and the decay is strongly incoherent at short times. Yet small-amplitude but long-lived oscillations are present in the occupations of both the donor and the acceptor up to ~ 1000 fs. They reflect weak vibrational coherence which survives longer on the picosecond time scale. This picture is in agreement with previously found results on an indocarbocyanine dye with a rather strong vibronic coupling (Duan et al. 2015a).

**Fig. 4:**
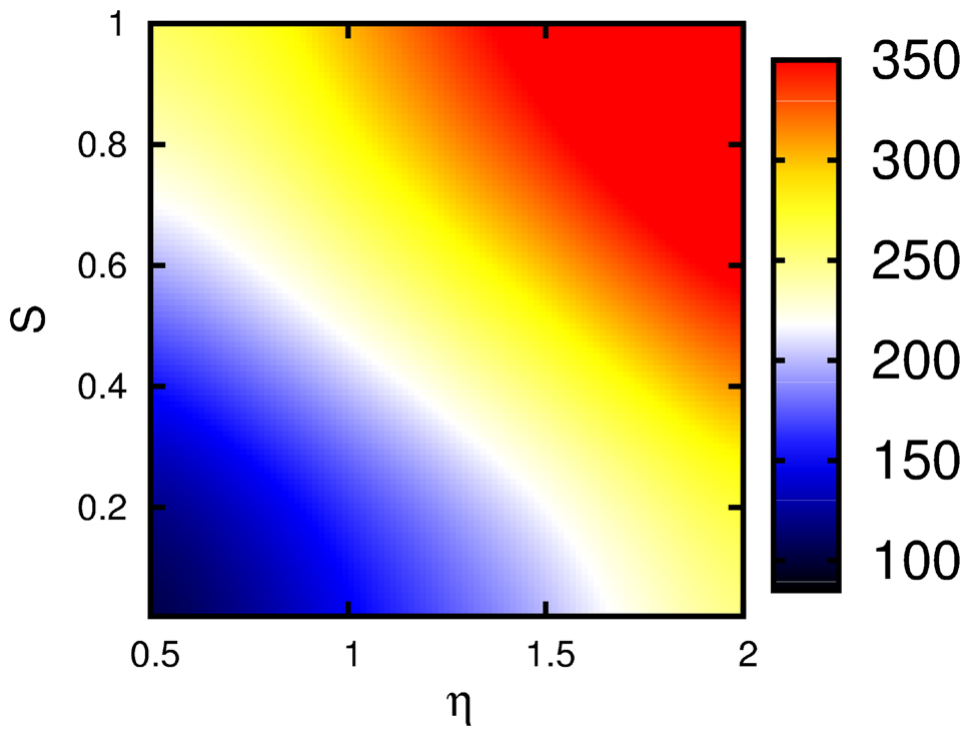
Transfer time of the population versus the system-continuous bath coupling strength $\eta$ and versus the Huang–Rhys factor *S* at temperature 300 K with $\gamma _{0}=10$ cm^−1^. The color bar shows the transfer time in fs

To study the impact of the underdamped vibrational modes on the exciton transfer more quantitatively, we consider the quantum yield of the transfer, which we identify with the population of the acceptor after 2 ps, where the steady state is reached within our accuracy. Also the vibrational coherence has died out. We plot this asymptotic acceptor population versus the Huang-Rhys factor *S* and system-background bath coupling strength $\eta$ in Fig. 3. In general, we find that the asymptotic occupation of the acceptor varies in a small range around 0.61–0.62 over the considered range of the Huang-Rhys factor and the system-bath coupling strength, rendering the acceptor occupation largely independent from these parameters. In Fig. 3, we see that for weak vibronic coupling, i.e., small S, the quantum yield is generally small and largely independent of the Ohmic damping constant *η*. When the vibronic coupling, i.e., the Huang-Rhys factor is increased, the quantum yield grows (see yellow region in Fig. 3). When *η* is increased for a fixed value of *S* (say $S=0.5$), the quantum yield decreases, illustrating that a large electronic damping hampers exciton transfer and leads to small quantum yields. Note that the case $S=0$ reflects a situation without a direct coupling of the states of the underdamped intramolecular modes.

**Fig. 5:**
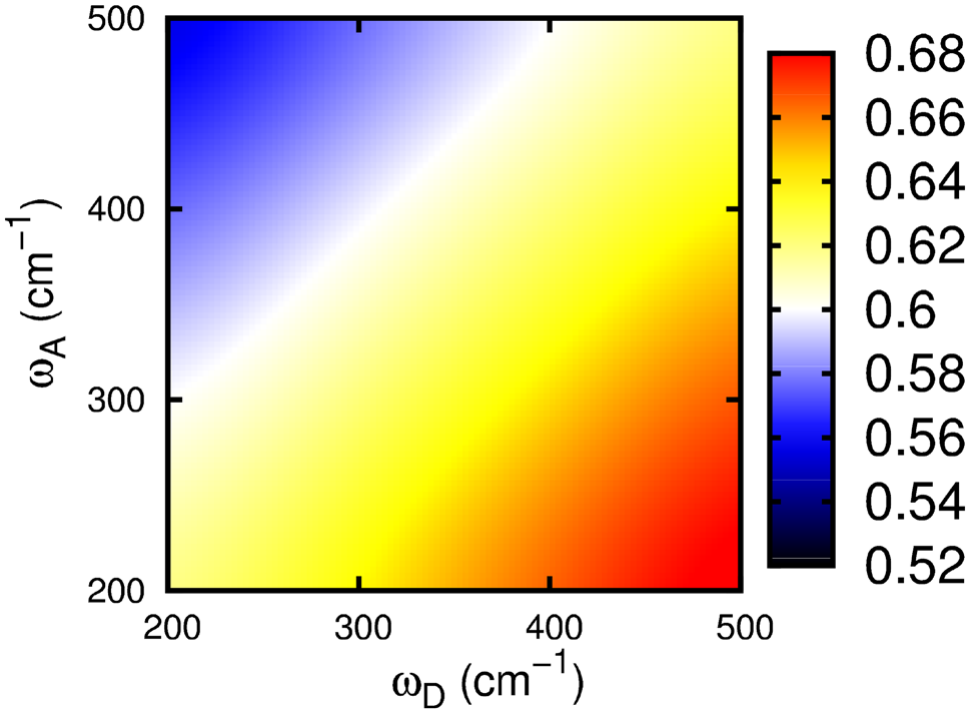
Quantum yield, or asymptotic population of the acceptor at $t=2$ ps, versus the vibrational frequencies of the modes at the acceptor $\omega _A$ and donor $\omega _D$. A Huang–Rhys factor $S=0.2$ was used with $\eta =0.5$ and $\gamma =200$ cm^−1^. The electronic coupling was assumed in the intermediate region $J=50$ cm^−1^

## Transfer time

Next, we analyze the time scale of the transfer of excitation from the donor to the acceptor. For this, we use a least square fit of the kinetics with an exponential function into the steady state by minimizing the residuals, for details see Duan et al. (2015b, 2015a, 2017). From this, we extract the time constant which we denote as the transfer time. Fig. 4 shows the transfer time for the same parameter region as for the quantum yield discussed above. It ranges from 100 fs in the weak coupling region, i.e., small system-bath coupling strength and small Huang–Rhys factor, to 350 fs in the strong coupling region with large system-bath coupling strength and a large Huang–Rhys factor. It is interesting to point out that the transfer is slower for a more strongly coupled vibrational mode or for stronger coupling to the incoherent background. Thus, within the dimer, the transfer of excitonic energy is slowed down.

We note that we have also studied a dimer with a stronger excitonic coupling with $J=150$ cm^−1^ and a vibrational frequency $\omega _{0}=316$ cm^−1^ (to study the same near-resonance configuration). We have observed the same qualitative behavior for this case as well (data not shown).

## Dependence on the vibrational frequency

Finally, we study a dimer where each monomer has an underdamped intramolecular mode with different vibrational frequency, i.e., $\omega _{D}\ne \omega _{A}$. We choose a fixed Huang-Rhys factor $S=0.2$, typical for the Fenna Matthews Olson complex (Wendling et al. 2000), $\eta =0.5$ , and $\gamma =200$ cm^−1^ in the following. The electronic coupling is again fixed in the intermediate region $J=50$ cm^−1^. We vary the vibrational frequencies of both intramolecular modes $\omega _{D}$ and $\omega _{A}$ in a range between 200 and 500 cm^−1^. Figure 5 plots the according quantum yield, i.e., the asymptotic population of the acceptor (again at $t=2$ ps). We find an overall variation of the quantum yield of roughly $\gtrsim 25$%, i.e., from 0.52 to 0.68. For equal vibrational frequencies of the donor and acceptor, no change occurs along the diagonal. Increasing $\omega _D$ of the intramolecular vibrational mode at the donor (at a fixed acceptor frequency) increases the final occupation of the acceptor because it is unfavorable to excite vibrational donor states in comparison to decay downhill to the acceptor state. This observation is in agreement with the analysis in the section “Mapping to a dissipative dimer-plus-oscillator system” and with Fig. 1, where the energy level scheme is shown. In the same way, decreasing the frequency $\omega _A$ of the intramolecular vibrational mode at the acceptor increases the final occupation of the acceptor because more vibrational acceptor states enter the energy transfer window. Maximal acceptor population of 0.68 is observed for maximal $\omega _D$ and minimal $\omega _A$ within our study. This is roughly a ~ 10% enhancement of the quantum yield compared to identical vibrational frequencies. Clearly, resonances between vibrational modes and excitonic transition energies are not relevant here.

## Conclusion

The impact of underdamped intramolecular local vibrational modes on the transfer of excitation energy in an excitonically coupled dimer model becomes apparent when considering the quantum yield and the transfer time. In a simple model of a dimer with two independent harmonic intramolecular modes, we find a significant enhancement of the asymptotic population of the acceptor when the acceptor interacts with an underdamped intramolecular mode with a frequency smaller than that at the donor. This statement can be generalized, since energy transfer from a light-harvesting complex to a weakly coupled nearby complex, i.e., the reaction center, is dominated by the weak coupling strength, as well as the effect of the pigment being coupled to the nearby complex, our result suggests an improved energy transfer efficiency between molecular complexes. Excitonic transfer within molecular complexes, however, slows down with increasing coupling to vibrational modes. Thus, underdamped vibrational modes which are weakly coupled to the dimer can fuel a faster transfer of excitation energy, if properly tuned. Note that when the vibrational modes at the donor and the acceptor have both equal frequencies, no enhancement of the quantum yield, i.e., of the final acceptor population, is found. The Huang-Rhys factor of the underdamped modes and the system-bath coupling strength do not modify this conclusion. Thus, transfer efficiency can in general be improved in nature when intramolecular vibrational modes are optimized. This result does not rely on long-lived electronic coherence.
